# Supplementary material for: Single Ventricle Reconstruction III: Brain Connectome and Neurodevelopmental Outcomes: Design, Recruitment, and Technical Challenges of a Multicenter, Observational Neuroimaging Study
Source: Diagnostics (Basel). 2023 Apr 30;13(9):1604. doi: 10.3390/diagnostics13091604 (PMC10178603; doi:10.3390/diagnostics13091604)
Supplement: Supplementary file 1 [file diagnostics-13-01604-s001.zip › diagnostics-2357085-supplementary.pdf]

Supplemental Table S1: SVRIII Brain Connectome Protocol: Harmonized Parameters for Siemens and Phillips 3T MRI

			Matrix	slices	FOV	% FOV phase	Resolution (mm)	TR (ms)	TE (ms)	TI (ms)	Flip Angle (deg)	Parallel Imagin g	Multi-band Acceleratio n	Phase partial Fourier	Diffusions Directions	b-values	Acquisition Time
Diffusion Imaging	Siemens	DTI	128 x 128	60	256 x 256	87.50%	2.0 x 2.0 x 2.0	8600	79	N/A	90	2x	Off	6/8	42	1000	7:27
		HARDI	100 x 100	60	240 x 240	100%	2.4 x 2.4 x 2.4	3300/3600	110/131	N/A	90	Off	3	6/8	64 x 4	3000, 5000	3:54 x 2, 4:16 x 2
	Philips	DTI	128 x 128	60	256 x 256	87.50%	2.0 x 2.0 x 2.0	6640	75	N/A	90	2x	Off	6/8	42	1000	7:31
		HARDI	100 x 100	60	240 x 240	100%	2.4 x 2.4 x 2.4	3033/3266	105/116	N/A	90	Off	3	6/8	64 x 4	2500, 4000	4:14 x 2, 4:46 x 2
Resting BOLD functiona l MRI	Siemens	rs-fMRI	64 x 64	36	256	100%	4.0 x 4.0 x 4.0	650	32	N/A	50	Off	4	6/8	N/A	N/A	5:09 x 2
	Philips	rs-fMRI	64 x 64	36	256	100%	4.0 x 4.0 x 4.0	800	32	N/A	90	Off	4	6/8	N/A	N/A	5:07 x 2
3D T1&T2	Siemens	T1	256 x 256	160	256 x 256	87.50%	1.0 x 1.0 x 1.0	2400	3.16	1200	8	2x	Off	Off	N/A	N/A	6:18
		T2	256 x 256	160	256 x 256	100%	1.0 x 1.0 x 1.0	3200	411	N/A	Variable	2x	Off	Off	N/A	N/A	3:30
	Philips	T1	256 x 256	160	256 x 256	87.50%	1.0 x 1.0 x 1.0	1870	3.3	900	8	2x	Off	Off	N/A	N/A	4:02
		T2	256 x 256	160	256 x 256	100%	1.0 x 1.0 x 1.0	2500	253	N/A	Variable	2x	Off	Off	N/A	N/A	4:28
